# A cluster randomized trial to evaluate external support for the implementation of positive behavioral interventions and supports by school personnel

**DOI:** 10.1186/1748-5908-9-12

**Published:** 2014-01-15

**Authors:** Ricardo Eiraldi, Barry McCurdy, Muniya Khanna, Jennifer Mautone, Abbas F Jawad, Thomas Power, Zuleyha Cidav, Jaclyn Cacia, George Sugai

**Affiliations:** 1 Department of Child and Adolescent Psychiatry and Behavioral Sciences, The Children’s Hospital of Philadelphia, 3440 Market St, Philadelphia, PA 19104-3306, USA; 2 Division of Developmental and Behavioral Pediatrics, The Children’s Hospital of Philadelphia, 3550 Market St, Philadelphia, PA 19104-3303, USA; 3 Department of Pediatrics, Perelman School of Medicine, University of Pennsylvania, 3401 Civic Center Blvd, Philadelphia, PA 19104-4319, USA; 4 Department of Psychiatry, Perelman School of Medicine, University of Pennsylvania, 3535 Market St, Philadelphia, PA 19104-3371, USA; 5 Devereux Center for Effective Schools, The Devereux Foundation, 2012 Renaissance Blvd, King of Prussia, PA 19406, USA; 6 Center for Behavioral Education and Research, Department of Educational Psychology, University of Connecticut, 249 Glenbroook Road Unit 2064, Storrs, CT 06269-2064, USA

**Keywords:** Implementation, Sustainability, Fidelity, Mental health services disparities, Behavioral health, Urban schools, School-wide positive behavioral interventions and supports

## Abstract

**Background:**

Urban schools lag behind non-urban schools in attending to the behavioral health needs of their students. This is especially evident with regard to the level of use of evidence-based interventions with school children. Increased used of evidence-based interventions in urban schools would contribute to reducing mental health services disparities in low-income communities. School-wide positive behavioral interventions and supports (SWPBIS) is a service delivery framework that can be used to deliver universal preventive interventions and evidence-based behavioral health treatments, such as group cognitive behavioral therapy. In this article, we describe our ongoing research on creating internal capacity for program implementation. We also examine the cost-effectiveness and resulting school climate when two different levels of external support are provided to personnel as they implement a two-tier SWPBIS program.

**Methods/Design:**

The study follows six K – 8 schools in the School District of Philadelphia randomly assigned to consultation support or consultation-plus-coaching support. Participants are: approximately 48 leadership team members, 180 school staff and 3,900 students in Tier 1, and 12 counselors, and 306 child participants in Tier 2. Children who meet inclusion criteria for Tier 2 will participate in group cognitive behavioral therapy for externalizing or anxiety disorders. The study has three phases, baseline/training, implementation, and sustainability. We will measure implementation outcomes, service outcomes, child outcomes, and cost.

**Discussion:**

Findings from this study will provide evidence as to the appropriateness of school-wide prevention and treatment service delivery models for addressing services disparities in schools. The effectiveness and cost-effectiveness analyses of the two levels of training and consultation should help urban school districts and policymakers with the planning and deployment of cost-effective strategies for the implementation of evidence-based interventions for some of the most common behavioral health problems in school children.

**Trial registration:**

ClinicalTrials.gov identifier: NCT01941069

## Background

### Services disparities

Several epidemiologic studies have shown that only one in five children with emotional and behavioral disorders receive mental health services [1-3]. Low-income and ethnically diverse children lag well behind their middle class, Caucasian counterparts in rate of service utilization [3,4]. Access barriers, such as lack of specialized services in low-income communities, high cost,and poor service quality, and stigma have been found to affect service utilization by ethnically diverse children [5-9]. Service delivery strategies that address access barriers and minimize the effects of stigma are likely to reduce service disparities [10].

### Schools’ role in reducing mental health disparities

Services provided in school settings are ideal for identifying and supporting children at risk for mental disorders [11] and for advancing the goal of reducing and eliminating services disparities because they are available to all children. Services are offered in convenient, close to home locations, are often provided at little or no cost to the families, and can be provided while the child is attending school [12-14]. School-based services reduce the stigma associated with seeking mental health services [15] and also afford the opportunity to serve children who are at risk for mental disorders [16-18]. As such, schools can play a significant role in addressing mental health services disparities in low-income urban communities [19,20].

### Externalizing and anxiety disorders

Aggressive, defiant, disruptive and antisocial behavior such as the behavior seen in children with, or at risk for, externalizing behavior disorders—*i.e.*, Oppositional Defiant Disorder (ODD), Conduct Disorder (CD)—have a lifetime prevalence of approximately 10% [21,22] and are highly prevalent in school settings [23-25]. These disorders have been found to lead to academic underachievement, grade retention, school suspension and expulsion, and later problems with the law [26-28]. Early onset of aggressive and antisocial behavior in elementary school has been found to be related to a persistent and chronic trajectory of antisocial behavior into middle childhood and adulthood [29,30].

Anxiety disorders—*i.e.*, Generalized Anxiety Disorder (GAD), Social Phobia (SP), Separation Anxiety Disorder (SAD)— affect up to 13% of the child population [31,32]. Anxious children are much more likely than non-anxious children to have problems with social and peer relations [33,34], academic achievement [35], school refusal [36,37] and future socio-emotional adjustment [38,39]. Children with GAD, SAD, and SP share the same underlying construct of anxiety [40,41], evidence a very high comorbidity rate and have been reported to respond similarly to treatment regardless of which disorder is principal [42,43]. Anxiety disorders are highly prevalent among inner city school children [44-46]. Urban children are especially at risk for anxiety because of the deleterious effects of living in unsafe and deprived neighborhoods [47].

### School-wide positive behavioral interventions and supports (SWPBIS)

SWPBIS is an integrated service delivery framework that targets changes in school climate by creating improved systems and procedures [48,49]. The practices and systems of SWPBIS are organized along a three-tiered continuum of prevention with a behavioral theoretical orientation and the empirical foundation of applied behavior analysis (ABA). Primary prevention strategies focus on preventing new cases of problem behaviors by using school-wide (Tier 1, universal) strategies, such as school-wide discipline, classroom behavior management, and effective instructional practices. Emphasis is placed on teaching all students key behavioral expectations and routines and creating a proactive means of communication for students and school staff. Randomized clinical trials have shown that schools employing SWPBIS have demonstrated better school climate (*e.g.*, fewer student disciplinary problems), higher levels of perceived school safety as reported by students and staff, greater reductions in the number of office discipline referrals (ODR), and improved reading scores compared to schools that did not use SWPBIS and/or did not implement SWPBIS with fidelity [48,50-54]. Some SWPBIS programs also offer targeted group-based support for at risk children (Tier 2, secondary prevention) and individualized support for more severe cases (Tier 3, tertiary prevention). In the present study, children who exhibit need for more targeted support will be offered participation in group cognitive behavioral therapy (GCBT) interventions for externalizing behavior problems or anxiety.

### GCBT for externalizing and anxiety problems

The Coping Power Program CPP; [55], a GCBT intervention, has been found to be effective at reducing aggressive behavior, covert delinquent behavior and substance abuse among aggressive boys [56]. Studies using a briefer version of CPP (Anger Coping) also reported significant reductions in aggressive behavior at post-intervention among targeted aggressive boys, compared to untreated aggressive boys and normal controls [57,58]. Another GCBT intervention, Friends for Life FRIENDS; [59], has been proven to be effective for the prevention and treatment of GAD, SAD, and SP [60-63]. For example, in a randomized trial with children diagnosed with an anxiety disorder, 76% of children assigned to FRIENDS were diagnosis free at the end of the 10-week trial compared to 6% of children assigned to a wait-list condition [63].

### Use of school personnel for implementation of evidence-based interventions (EBIs)

A number of recent studies have shown that school personnel can be successfully trained in the development and implementation of SWPBIS [48,50,64] and GCBT for externalizing and anxiety disorders [65-68], respectively. Also, SWPBIS has been successfully implemented in a number of inner city schools around the country [69,70]. Unfortunately, school personnel and school counselors often lack adequate training in the implementation of SWPBIS and other EBIs. In addition, schools in urban settings often do not have adequate funding to contract mental health professionals to provide child services. The present study directly addresses these important barriers by enhancing school capacity to deploy EBIs.

### Theoretical framework

**Figure 1:**
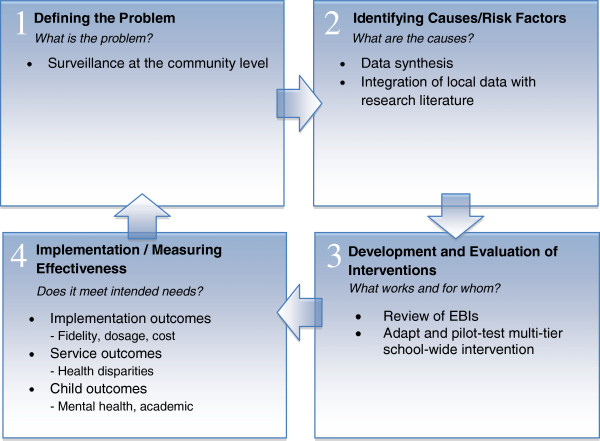
**Public health model for project development and implementation.** Adapted from Kutash, K., Duchnowski, A. J., & Lynn, N. (2006). School-based mental health: An empirical guide for decision-makers. Tampa, FL: University of South Florida, The Louis de la Parte Florida Mental Health Institute, Department of Child & Family Studies, Research and Training Center for Children’s Mental Health.

We developed and pilot-tested the program in two K–8 schools that have the same ethnic and socio-economic breakdown as the schools participating in this study. The project was funded by the Centers for Disease Control and Prevention (CDC; RFA-CD-08-001; *Elimination of health disparities through translation research; R18*). The adaptation and testing of the interventions used in the study were organized around the first three steps of the Public Health Model (*i.e.*, Defining the problem; Identifying causes/risk factors; Developing and testing the intervention) See, [71-73] (see Figure 1). Specifically, we collected needs assessment data, assessed symptom profile and mental health service utilization of typical students in the participating schools, investigated risk and protective factors, conducted a review of EBIs for ethnically-diverse children, selected and adapted interventions based on the characteristics of the student population and specific constraints of under-resourced schools, and pilot-tested SWPBIS and EBIs for externalizing behavior problems and anxiety (Eiradi et. al., Development and implementation of a SWPBIS program with mental health supports in urban schools, submitted) [74]. The present study will extend this work by addressing the fourth step (Implementing the intervention/measuring effectiveness) of the Public Health Model [71]. Outcome measures for the present study were selected based on the implementation framework developed by Proctor and colleagues [75,76], including the assessment of implementation outcomes, service outcomes, and child outcomes (see Figure 1).

### Study aims

The specific aims of the study are:

1. To determine whether program content and process fidelity for Tier 1 (for all students) and Tier 2 (for at risk and high risk students) differ between schools receiving a higher level of support (Consultation plus Coaching, C + C) and schools receiving a lower level of support (Consultation, C) for program implementers.

2. To determine whether school climate, office discipline referrals, and participants’ diagnostic status, symptom and impairment severity, coping skills, and academic productivity differ between schools receiving different levels of support (C or C + C).

3. To assess changes in mental health disparities brought about by Tier 1 (perception of school climate, student suspension rates) and Tier 2 (proportion of students with unmet need), respectively.

4. To determine the incremental cost-effectiveness of SWPBIS C versus SWPBIS C + C.

### Study fit with the funding opportunity

The study is aligned with the funding opportunity *PAR-11-104, Reducing health disparities among minority and underserved children (R01)*, in that it evaluates the comparative effectiveness of two levels of support for school personnel for the implementation of a continuum of mental health prevention strategies for low-income, ethnically diverse children in non-traditional service settings.

## Methods/Design

**Figure 2:**
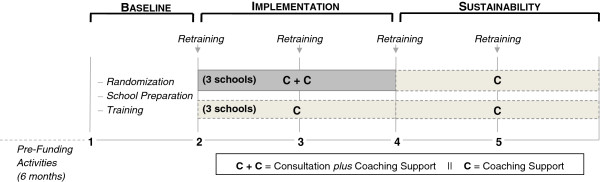
Study design and timeline.

Six K–8 schools in the School District of Philadelphia were randomly assigned to either Consultation or Consultation plus Coaching (See Figure 2). A stratified random assignment list of schools to condition was generated using a computerized random assignment program [77]. Schools were stratified to group one if their baseline School Climate (CREST) scores were below the median CREST scores or to group two if the CREST scores ≥ the median value. The units of analyses for Tier 1 are all of the students in those K-8 schools (650 per school), leadership team members (eight per school) and other school staff (30 per school). The units of analyses for Tier 2 are the children meeting criteria for inclusion in one of the GCBT groups (enroll a total of 367 students assuming a 17% dropout; approximately 51 eligible students per school), the school counselors (two per school) conducting the GCBT groups, and the groups themselves (14 per school; see Table 1).

**Table 1:** Participants by Tier and support level

		**Consultation**	**Consultation plus coaching**	**Total**
**Tier 1**				
	Students	1,950	1,950	3,900
	Leadership team members	24	24	48
	School staff	90	90	180
**Tier 2**				
	Students	153	153	306
	Counselors	6	6	12
	Intervention groups	42	42	84

### Participants

Tier 1 research activities will include 48 teachers, administrators and parents in the leadership teams, 180 school staff for the assessment of school climate, and approximately 3,900 students. Participants in Tier 2 will be 12 counselors and approximately 306 evaluable children who have, or are at risk for, externalizing or anxiety disorders. School personnel will provide all services. Information pertaining to each school will be considered nested within school ‘cluster’ and will be defined as such in the statistical analyses.

### Inclusion criteria

All children will participate in Tier 1. Children in grades 4–8, who meet screening and diagnostic criteria will be included in Tier 2. The screening criterion is a score ≥1 SD on the Conduct Problems or Emotional Symptoms scales of the Strengths and Difficulties Questionnaire (SDQ), a widely-used screening instrument [78], filled out by a teacher. Children meeting screening criterion will undergo a diagnostic evaluation. Children meeting primary diagnosis of ODD, CD, GAD, SAD, or SP (based on a parent structured interview [79] and a symptom severity scale [80] filled out by an independent rater) at Intermediate or Positive level will be eligible for participation. Children with comorbid (secondary) conditions will be included.

### Exclusion criteria

There are no exclusion criteria for Tier 1. Children with a Special Education classification of Intellectual Disability, as well as those who are not able to communicate in English, do not have a principal diagnosis of ODD, CD, SAD, GAD or SP or are not at risk for one of those disorders, have an accumulated absenteeism record of ≥33% for the current year, or have a history of psychotic or autistic spectrum disorders, will be excluded from Tier 2.

### Linkage between Tier 1 and Tier 2

The leadership teams and the counselors will manage the SWPBIS program. They will be in charge of identifying, eliminating, or consolidating existing school-wide interventions that could overlap or interfere with the implementation of SWPBIS. Existing behavioral health services for individual students (*e.g.*, wrap-around services) will be maintained because they will not interfere with SWPBIS. The leadership teams in each school will develop all interventions for Tier 1 while receiving C or C + C. A subcommittee within the leadership team composed of a counselor and an administrator will be in charge of identifying and referring children for participation in Tier 2. Following methods used in other studies [81] each teacher will be asked to rank the children in their classroom from least to most difficulty with externalizing behavior or anxiety and then to refer the top three children with externalizing behavior problems and top three children with excessive anxiety for possible participation in Tier 2. Decisions as to the appropriateness of referrals to Tier 2 will be based on data gathered via teacher ratings on the SDQ [78] and a web-based software system [82] for collecting and summarizing office discipline referrals.

### Setting

The SWPBIS project will be implemented in six K–8 public schools in North Philadelphia, PA, a low-income area that has the second highest level of food insecurity in the country [83]. The ethnic makeup of students is approximately 60% Latino, 30% African American, 10% other minority.

### Training and consultation to leadership teams and counselors

The training model assumes that participating school personnel will need initial training during the first year of the project and briefer retraining each subsequent year. Members of the research team will conduct three days of formal initial training and one day of retraining each with members of the leadership team (LT) and counselors. The training will be delivered using procedures employed in dissemination and implementation studies in nontraditional settings (*i.e.*, initial workshop and subsequent ongoing consultation) [68,84] and other strategies found to be effective according to a recent meta analysis (*i.e.*, active learning strategies) [85].

### External coaches

The support conditions (C, C + C) were modeled after procedures developed by John Lochman and colleagues for the training of school counselors in the context of a dissemination study with the Coping Power Program [68] and recommendations from a recent meta analysis [85]. Consultation will be provided by ‘coaches’ who will be advanced trainees (*e.g.*, interns, fellows) in applied psychology. After initial training, which will be the same for C and C + C, the Tier 1C + C Coaches will conduct one-hour biweekly on-site consultation with members of the LT to help them develop and implement universal interventions throughout the school as delineated in the SWPBIS Team Training Manual [86]. Progress toward the completion of each step in the SWPBIS Team Training Manual will be assessed monthly via the Team Implementation Checklist (TIC) and PBIS Action Plan [87] completed by members of the LT and reviewed by the Tier 1 Coach. In addition, the Tier 1 Coaches will be available to the head of the LT for scheduled one-on-one performance feedback and as needed one-on-one consultation (via telephone or email) about implementation barriers (Individualized Problem Solving). The Tier 1C Coaches will supply the LT an online guide containing steps for the development and implementation of universal interventions. Also, the Tier 1C Coaches will have one-hour biweekly monthly phone conferences with the head of the LT in which progress in completing the steps needed for the development and implementation of interventions as measured via the TIC and PBIS Action Plan will be discussed.

After the initial training related to Tier 2 interventions, which will be the same for C and C + C counselors, the Tier 2C + C Coaches will conduct 14, two-hour weekly on-site consultation sessions with counselors to debrief previous CPP and FRIENDS sessions, observe child group sessions, prepare for upcoming sessions, and conduct problem solving concerning barriers and challenges in the implementation of the protocols. Counselors will receive video-recorded samples of effective implementation of the main components of the treatments (*e.g.*, exposure for anxiety). Tier 2C + C Coaches will also provide individual performance feedback to the counselors after the session is over and will be available to counselors as needed (via telephone or email) for one-on-one consultation about implementation barriers (Individualized Problem Solving). Tier 2C Coaches will conduct two-hour weekly consultation sessions using the same group content and format and they will provide counselors video-recorded samples of effective implementation of the main components of the treatments. However, they will not observe group sessions or offer individualized feedback or individualized support to counselors.

### School recruitment

Upon receiving funding, a presentation of the project was conducted with a preselected group of school administrators from schools in the regional area of interest. Presentations were conducted in person and via GoToMeeting. Those who expressed interest in taking part in the project were asked to respond to a brief request for proposals (RFP). The six schools that applied met the minimal inclusion criteria, so the search was discontinued at this stage. The inclusion criteria included: grade level (any elementary or middle school combination); students’ socio-economic status (percentage of students eligible for free/subsided lunch set at ≥ 90%); racial/ethnic diversity (majority of students are of minority status); and school-wide initiatives (absence of current mental health prevention initiatives). A more in-depth description of the project was provided during staff meetings and a vote was taken. At least 80% of school staff in attendance was required to vote affirmatively in order for the school to join the project. This is a standard cut-off in SWPBIS [88]. Subsequently, the investigators trained members of the leadership teams. As of this writing, the leadership teams are developing interventions for Tier 1 while receiving C or C + C.

### Measures

We employ two measures to help us determine the readiness of school personnel for implementing Tier 1 and Tier 2 interventions. Members of the leadership teams will complete a 23-item checklist [87] to assess completion of activities related to critical features of the SWPBIS model, including regular meetings of the LT and the use of discipline data (*e.g.*, events, dates and time, student, in and out of school suspensions) for program planning and to monitor the schools’ progress in implementing their SWPBIS Action Plan [48]. Members of the leadership teams will complete the instrument monthly; it will also be used by the External Coach to guide members of the LT in developing and implementing Tier 1 interventions in their school.

For Tier 2, following the initial workshop, trainers will assess whether counselors have learned the theoretical underpinnings of CBT and the treatment of anxiety and depression in children. We will use a questionnaire [89] developed for the assessment of knowledge of EBIs in the treatment of youth psychopathology. Counselors will complete the questionnaire right after the initial and last workshop. Counselors scoring below the 80% cutoff will be provided further individual training in the areas in which they scored low.

### Screening, demographics, service utilization

Counselors will ask teachers to complete the SDQ, a mental health screening questionnaire [78,90,91], for children who accumulate ≥3 ODRs or who are referred by a subcommittee of the LT charged with monitoring at-risk children. Children who score ≥1 SD [92] on the Conduct Problems or Emotional Symptoms scales will be invited to participate in a thorough diagnostic assessment.

### Implementation outcome measures

#### Fidelity

Delivering content as originally intended by the treatment developers (content fidelity) leads to better outcomes [93]. Also, delivering content while actively engaging clients in therapy and doing it with a sense of competence (process fidelity) leads to better adherence and outcomes [94-97]. A group of 20 randomly selected teachers, other school personnel and students will be interviewed at the end of each project year in order to assess content fidelity for Tier 1 [98]. Data sources are direct observations, review of school policies and interviews with school staff and students [99]. A cut-off score of 80% on the measure will be used for determining successful implementation of SWPBIS [98].

A checklist reflecting each activity component of the session agenda or outline for CPP and FRIENDS will be used to assess content fidelity for Tier 2. All treatment sessions will be video recorded. Independent coders (ICs) will use a yes-no response scale to indicate whether or not a counselor covered a particular component. Two ICs will complete the checklist after observing video recorded sessions. Differences of within 5% between the 2 ICs will be considered an agreement. We will report the average score between the two ICs.

Our process fidelity checklist is based on a 12-item measure developed by John Lochman and colleagues (*e.g.*, Counselor’s tone is warm and positive). We will use the total score (Overall Process Fidelity) [68]. Twenty percent of sessions one, two, three, and four will be coded, while 30% of sessions five through 14 will be coded by 2 ICs. Differences of within one point between the two ICs will be considered an agreement. We will report the average score between the two ICs.

#### Process evaluation

We will conduct focus groups with members of the leadership teams and other school personnel at the end of each project year in order to assess perceptions of the appropriateness of C and C + C level of support for the implementation of SWPBIS.

#### Children’s functioning

We will collect children’s academic performance, absenteeism, and office discipline referrals (ODR) throughout the five years of the project. Absenteeism data will be collected from the school’s daily attendance records. Academic performance will be taken from the state’s mandatory testing for reading and math for all students. ODRs will be collected using a web-based system for monitoring ODRs to assist in intervention planning and evaluation [100]. These data will be used for determining inclusion into Tier 2 and to measure outcomes. We will also record in- and out-of-school suspensions per 100 students per year and percentage of students receiving suspensions. These indices are reliable and valid for measuring intervention effects [82].

#### School climate

Perception of school climate will be assessed via a questionnaire [101] completed by 30 school staff per school (equal proportion of teachers, support staff and administrators) chosen at random in year one. The same school staff in each school will subsequently complete the survey at the end of each project year. The questionnaire has four factors: Skill Instruction; Support for Staff; Staff Respect for Students; and Safety. We will use all four factors in the analyses.

#### Mental health disparities

We will measure changes in mental health disparities as a result of Tier 1 interventions by comparing scores on school climate and school suspension rates between year one (baseline) and each subsequent year across all schools. Changes in mental health services disparities as a result of Tier 2 interventions will be assessed via a semi-structured service utilization interview [102,103] administered to the parents of children in Tier 2 across all schools between the baseline and intervention phases.

### Child outcome measures for at risk/high risk children

Children’s diagnostic status, mental health symptoms, coping skills and functional impairment will be assessed at pre- and post-participation in CPP and FRIENDS. All of the parent measures are available in Spanish. Diagnostic status will be assessed via a computerized parent-structured interview [79,104]. This instrument reports three levels of diagnostic severity for each disorder: Positive, Intermediate (at-risk), or Negative. Upon completion of the structured interview, the independent evaluator (IE) will assign interference scores to each positive diagnosis in order to determine primary and secondary diagnoses [80], based on a seven-point scale, with lower scores indicating less severity. Changes in coping skills will be assessed via a 34-item self-report questionnaire [105] that measures coping strategies (seeking social support, self-reliance/problem-solving, distancing, internalizing, and externalizing). The total score for this measure will be used in outcome analyses.

Changes in mental health symptoms (*e.g.*, aggression, conduct problems, anxiety, somatic symptoms) will be measured via parent [106], child [107,108] and teacher [106] multi-axial rating scales. Teachers will also provide changes in children’s grades and rate of academic productivity [109]. After all parent, child and teacher data are collected at pre- and post-, the IEs will complete a measure of functional impairment [110].

### Independent evaluator (IE) training and reliability

IEs will be advanced doctoral students in applied psychology who have been trained to a reliable standard on the use of diagnostic instruments by M.K. and J.M. following recommended guidelines through joint interviews, live observation, and discussion during the pre-study start-up meetings. IE reliability will be checked quarterly. Kappa coefficients will be computed, and a minimum of 0.85 will be required for each measure.

### Interventions

#### Primary prevention (universal interventions)

The use of SWPBIS in this study differs from other studies in that it focuses primarily on student mental health (*i.e.*, emotional and behavioral functioning). The focus of primary prevention strategies will be to prevent new cases of mild externalizing or anxiety problem behaviors by using school-wide (universal) strategies. A key intervention will be the development of a leadership team. The primary responsibility of the leadership team will be to develop, implement and formatively evaluate Tier 1 interventions. In the process, a set of school-wide expectations will be developed, explicitly taught to all students and promoted through the implementation of a school-wide motivation system. Specific rules and positive and negative consequences for student behavior in hallways, cafeteria, and playgrounds will be developed, taught to students, and implemented and monitored by school personnel. We expect that Tier 1 interventions will lead to improved school climate and perceived school safety. Improved school climate and perceived school safety will likely contribute to a decrease in the number of children who develop externalizing behavior problems, and also, anxiety problems. We will have two tiers in the proposed study: Tier 1 for universal interventions and Tier 2 for children who meet diagnostic criteria or are at risk for an externalizing or an anxiety disorder.

#### Secondary prevention (at-risk/high risk students)

The Coping Power Program (CPP) is a cognitive-behavioral, multi-component group intervention for elementary and middle school students at risk for externalizing behavior disorders. In addition to anger management, the CPP includes units on goal setting, emotional awareness, relaxation training, social skills training, problem solving, and handling peer pressure. The original CPP offers eight sessions for the first year of intervention and 25 sessions for the second year of intervention. We adapted the protocol based on qualitative (focus groups) and quantitative (acceptability) data from parents, children and teachers. We reduced CPP from the original 34-session program to a 14-session format to make it possible for counselors in busy under-resourced urban schools to run groups with CPP. Care was taken to maintain the key components (active ingredients) of the treatment, even though less time was dedicated to covering each Section. A previous shorter version of CPP (Anger Coping Program) has been found to be very effective with a group of aggressive boys [58,111].

Friends for Life (FRIENDS) is a group CBT intervention, based on a theoretical model, which addresses cognitive, physiological and behavioral processes that are seen to interact in the development and perpetuation of excessive anxiety. The original FRIENDS protocol consists of 10 (60-minute) weekly sessions and two booster sessions. In the present study, we have included the booster sessions in the regular protocol and added two more sessions in order to fit them into the typical class period (40 minutes), for a total of 14 sessions. The protocol has a parent component, which consists of two group sessions that focus on strategies to help parents cope with their own anxiety, reinforcement strategies and contingency management for children, and brief training in problem solving and communication skills.

### Data analytic plan

The statistical analyses for each of the study aims are described below: in addressing aim one, mean fidelity and its 95% confidence intervals for Tier 1 will be calculated using 20 randomly selected participants from each of the six schools during years three, four, and five (120 total per year). The 95% confidence intervals will be presented by C & C + C for all schools combined and for each school. Content and process fidelity data for Tier 2 will be obtained by scoring 14 video recorded sessions per each of the treatment groups. Perception of School Climate (aim two) during year one will be surveyed by a randomly selected sample of teachers, support staff and administrators from each participant school. The randomly selected sample will consist of similar proportion of teachers, support staff and administrators within each school. The questionnaire [101] will be completed at the end of years one, two, three, four, and five. This 31-item, 5-point Likert type scale checklist has four factors. Each factor’s total score will be analyzed separately. School personnel no longer present in the school during subsequent years will be replaced.

The general analytical approach for testing our hypotheses will be the Laird and Ware mixed effects model [112] and the Generalized Estimating Equation (GEE) approaches [113,114]. Mixed effects and GEE are statistical approaches based on regression techniques for analyzing correlated data collected repeatedly from the same subject. The study design will allow us to examine the between- subjects effect related to the two levels of support, and a within-subjects effect corresponding to time points (five time points), as well as a type of support (C, C + C) by time interaction effect. These analyses will be conducted using SAS Proc Mixed, which utilizes the mixed effects models, and SAS Proc Genmod, which utilizes GEE [115]. Both the mixed effects model and GEE approaches allow estimation of fixed effect parameters such as time, and time by treatment interaction. If the interaction term is significant, it indicates that change in school climate and/or reduction in ODRs over time is significantly different between the two levels of support. If the interaction term is statistically not significant, the changes over time for the two primary outcomes will be presented by the time effect only, which represents both levels of support. The advantage of utilizing these methods is that all information available from each student is used, including variables with missing observations. In the proposed analyses, we will consider students measured within classes are correlated and will be nested within classes. Nested analysis will be chosen as part of Proc Mixed and Proc GEE, as such, the intraclass ‘intracluster’ correlation (ICC) will be part of the overall variance covariance matrix.

Changes from pre- to post- implementation in diagnostic status, symptom severity and impairment level, coping skills and academic productivity during Implementation across all schools, regardless of level of support, will be analyzed using McNemar tests. For each of the listed primary outcomes, a series of 2X2 tables will be constructed to test changes from pre- to post- in the pertinent categories using McNemar tests. Alpha level will be adjusted to 0.025 based on Bonferroni criteria for multiple comparisons. We will also use McNemar tests to test the hypothesis that the pre- to post- level of improvement in diagnostic status, symptom severity, impairment level, coping, and academic productivity obtained during Implementation will be maintained during Sustainability among schools receiving C + C.

We would like to determine whether pre- to post- improvement in diagnostic status, symptom severity, impairment level, and academic productivity differ by support condition during the implementation phase. In addressing this research question, and for the sake of simplifying the statistical analysis, each student will be categorized as either improved or not improved based on change from pre- to post-. A category one will be assigned if a student’s diagnostic status, for example, changes either from positive to intermediate, intermediate to negative, or positive to negative. Otherwise a score of zero will be assigned. Similar coding will be used for symptom severity, coping, impairment level, and academic productivity. We will use chi-squared tests to compare the proportion of (yes/no) for each of the primary outcomes between C and C + C type of supports. To examine changes in the rate of ODRs between baseline and follow up during each school year, the number of students with at least one ODR will be defined at baseline and at the follow up and a 95% confidence interval for such changes will be constructed for each of the five years.

In addressing aim three, school climate scores and the proportion of out-of school suspensions (number of suspension divided by number of students) served at the school during the previous academic year (*i.e.*, before implementation of Tier 1) will be compared to school climate scores (using the two independent sample t-test), and school suspensions for each subsequent project year (using Chi-squared test) respectively. For Tier 2, the proportion of children at baseline who are found to have unmet need for mental health services (*e.g.*, children found to have an externalizing or anxiety disorder at the intermediate or clinical level via the DISC-IV and who have not received mental health services as measured via the SACA) will be compared to the proportion of children with unmet need for mental health services in subsequent years (using Chi-squared test). In order to obtain a measure of unmet need at baseline, we will interview parents to assess the presence of disorders and service utilization at one year prior to the assessment and at the time of the assessment. For each subsequent cohort of children, we will interview parents about presence of disorder and service utilization only at the time of the assessment. Comparison of the proportion of unmet need between each subsequent year to the baseline proportion will be compared using Chi-square test.

### Aim four: cost analysis

We will evaluate the effectiveness of SWPBIS with C + C as compared to SWPBIS with C in increasing procedural and process fidelity and in reducing ODRs and improving school climate, and children’s grades, diagnostic status, symptom severity and impairment; and the effectiveness of SWPBIS with C + C as compared to SWPBIS with C. An incremental cost-effectiveness ratio [116] will be constructed for each intervention (SWPBS with C + C for Tier 1, SWPBS with C for Tier 1, SWPBS with C + C for Tier 2, SWPBS with C for Tier 2). The denominator of the cost-effectiveness ratio is the difference between the effectiveness of the intervention and control groups (accounting for baseline levels) on designated measures of fidelity and student outcomes (*e.g.*, student grades). The numerator of the cost ratios is the difference in mean costs for intervention and control groups (accounting for baseline levels). All costs associated with the program will be set at $0 at baseline. Costs for interventions will include: initial development of the leadership team; initial training of the coaches (Tier 1) and clinical supervisors (Tier 2); initial training of the leadership team and school counselors; subsequent supervision of coaches and clinical supervisors; conducting groups with at risk children; and activities to maintain the SWPBIS program, including day-to-day implementation, ongoing training, data collection, and money for student incentives. Within each component, two main types of costs will be calculated: cost of physical materials used for training and interventions and costs associated with time spent by trainers and school personnel [117].

As an example, the cost-effectiveness ratio for fidelity will reveal how much it costs (or how much it saves) to increase fidelity among the study population by one point. Confidence intervals will be constructed to determine the probability that the difference between two ratios indicates significant differences in cost-effectiveness for the target population. The ratios can then be ordered from lowest to highest to show which intervention is the most cost-effective for increasing the SET score. Administrators then have the information they need to determine if gains in fidelity are sufficient to justify marginal costs. In a similar manner cost-effectiveness ratios can be constructed for other targeted outcomes (*e.g.*, measures of school climate, child symptomatology, impairment, coping skills, symptom severity, and functioning) for comparisons in which the results indicate significant improvement in the intervention group as compared to the control condition.

### Examination of focus group results

As indicated, focus groups will be conducted with LTs to identify their perceptions of SWPBIS implementation in their schools. Sessions will be transcribed and analyzed qualitatively, based on grounded theory. Two RAs will read the transcripts independently and record all major themes on index cards. The RAs will present the data to the research team. The research team will then try to resolve any discrepancies between the RAs’ findings. Next, the RAs will examine the transcripts once again and identify the frequency of comments pertaining to each theme. The frequencies for RA one and RA two will be compared to check for inter-rater agreement. Standard frequency tables will be used to compare the relative frequency of endorsements for each theme. The agreement between the two RAs will also be checked by calculating the Kappa co-efficient.

### Sample size justification and statistical power

Sample size estimation was based on addressing the primary outcome, the participants’ diagnostic status (aim two). The statistical analysis will be McNemar tests for two correlated proportions (pre/post) between two groups. With a sample size of 300 students, the statistical test achieves 92% power to detect an odds ratio of three using a two-sided McNemar test with a significance level (α ) of 0.01. The odds ratio is equivalent to a difference between two paired proportions of 0.25, which occurs, for example, when the proportion among students diagnosed with a Positive anxiety/externalizing disorder at pre and improved to Intermediate at post (cell 1,2 in the 2X2 McNemar table) is 0.425 and the proportion among students diagnosed without Positive anxiety/externalizing disorder who became either Intermediate or Positive at post (cell 2,1 in the 2X2 McNemar table) is 0.175. Therefore, the proportion of discordant pairs is 0.60. We adjusted α level to 0.01 to account for the multiple 2X2 McNemar testing. We need to recruit six schools, grades four to eight,to be able to enroll 300 evaluable students who meet inclusion criteria. We assumed that the observed changes in students’ diagnostic status within schools are independent based on an evaluation of the FRIENDS program, in which ‘schools’ level accounted for less than 5% of the total variance across dependent measures.

## Discussion

### Innovation

The study is innovative in a number of ways. This is the first study utilizing SWPBIS as a foundation for addressing mental health disparities in urban schools and one of the first studies embedding EBIs for both externalizing and anxiety disorders within a SWPBIS framework. With regard to implementation science, this is the first study assessing the effectiveness of two randomized levels of support for school personnel implementing EBIs. The study uses rigorous methods for the measurement of intervention content and process fidelity within a two-tier mental health program. We will also conduct a cost analysis and measure the effects of Tier 1 and Tier 2 interventions on academic productivity, grades, attendance, disciplinary referrals, and suspensions, which are rarely assessed in this type of dissemination and implementation study [12].

### Challenges and limitations

Implementing SWPBIS with mental health supports in under-resourced urban schools presents a number of challenges. In our pilot study where we developed and piloted the interventions, we faced repeated leadership changes at the district level, staff turnover at the school level, and limited parent participation [74]. However, we also found strong and consistent support among second-level administrators (*e.g.*, regional superintendents) at the District level and committed leadership teams that were able to recruit new members who functioned effectively. With the lessons learned from our pilot study, we are able to more effectively support the schools in successfully implementing SWPBIS while receiving either level of support. We have redoubled our efforts at helping schools to increase parental collaboration and support, which lead to more parent participation. We have also designed the implementation strategy to fully utilize existing policy and infrastructure (*e.g.*, implementation of District-wide practices to promote students’ success; utilizing school counselors and school facilities for Tier 2 interventions) to limit strain on resources so that the proposed program will be sustainable.

A limitation is that we are conducting the study in six schools within a single school district (though it is one of the largest and most diverse school districts in the country), which limits generalizability to schools in different parts of the country. Including a relatively small number of schools limits our ability to examine the clustering effect of school classes and schools. In this study, the unit of randomization is the school but the unit of analysis (aim two) is the student. A cluster randomized approach would have been an appropriate design if a much larger number of schools were included in the study. However, it is anticipated that the degree of similarity in treatment response among students within a class will be poorly correlated, and therefore the intraclass correlation coefficient within clusters will be small and its effect on the overall variance between schools would be minimal. However, an appropriate cluster analyses will be conducted using nested analysis. The NESTED Procedure of SAS [115] is an example of such analysis. We expect that our findings will inform implementation research in schools and procedures to be incorporated into larger-scale trials in urban schools in the future.

## Conclusions

This study has the potential to show the amount of resources (training and consultation) needed for the implementation of EBIs with high levels of fidelity in under-resourced urban schools. It will also begin to show the type of resources needed in order to sustain this type of program longer term. The cost and cost-effectiveness analyses will enable urban school districts and policy makers to determine costs to successfully deploy SWPBIS with integrated mental health supports in each additional school and throughout the district and how much additional support and cost would be needed in order to improve implementation fidelity, and school and child outcomes.

## Trial status

ClinicalTrials.gov identifier: NCT01941069. The study was approved by the Committee for the Protection of Human Subjects of The Children’s Hospital of Philadelphia and the Devereux Foundation (IRB 12–009477) and by the Office of Research and Evaluation (ORE) Research Review Committee of the School District of Philadelphia (IRB 2012-07-099).

## Abbreviations

ABA: Applied behavior analysis; CBT: Cognitive behavioral therapy; CD: Conduct disorder; C: Consultation; C + C: Consultation plus coaching; CPP: Coping power program; EBI: Evidence-based intervention; FRIENDS: Friends for life; GAD: Generalized anxiety disorder; ODD: Oppositional defiant disorder; SAD: Separation anxiety disorder; SP: Social phobia; SWPBIS: School-wide positive behavioral interventions and supports.

## Competing interests

The authors declare that they have no competing interests.

## Authors’ contributions

RE conceived the study, drafted the manuscript and approved all edits. BMC, MK, and TP collaborated on the design of Tier 1 and Tier 2 interventions. AJ prepared all quantitative analyses. ZC prepared the cost analyses. JM serves as project director. JC serves as project coordinator and IRB regulatory. GS is a consultant on this project. All authors reviewed and edited the manuscript. The final version of the manuscript was vetted and approved by all authors.
